# Effect of Anti-Clouding Agent on the Fate of 3-Monochloropropane-1,2-Diol Esters and Glycidyl Esters in Palm Olein during Repeated Frying

**DOI:** 10.3390/molecules24122332

**Published:** 2019-06-25

**Authors:** Azmil Haizam Ahmad Tarmizi, Raznim Arni Abd Razak, Abdul Niefaizal Abdul Hammid, Ainie Kuntom

**Affiliations:** Malaysian Palm Oil Board, 6, Persiaran Institusi, Bandar Baru Bangi, Kajang 43000, Selangor, Malaysia; raznim@mpob.gov.my (R.A.A.R.); niefaizal@mpob.gov.my (A.N.A.H.); ainie@mpob.gov.my (A.K.)

**Keywords:** polyglycerol fatty acid esters, 3-monochloropropane-diol-1,2-diol esters, glycidyl esters, palm olein, intermittent frying conditions

## Abstract

Issues on 3-monochloropropane-diol-1,2-diol (MCPD) esters and glycidyl esters in refined oil have gained much attention when these heat-induced contaminants are associated with health implications. Oil that undergoes the frying process could influence the fates of 3-MCPD esters and glycidyl esters, especially with the addition of an anti-clouding agent. In this study, we investigated the effect of polyglycerol fatty acid esters (PGE) on the transients of 3-MCPD esters and glycidyl esters in palm olein (POo) during intermittent frying. Thermal resistance of POo fortified with PGE (0.1% to 0.4%) was assessed for 8 h of daily frying operations at 180 °C across five consecutive days. The addition of PGE decelerated the reduction of 3-MCPD esters and glycidyl esters with the progression of frying. The presence of these compounds coincided with the amount of oil taken up by the fried product. The inclusion of PGE in POo also induced higher augmentation of polar compound fractions, i.e., oxidised triacylglycerols (OxTAG) and polymerised triacylglycerols (PTAG), but gave comparable free fatty acid (FFA), *p*-anisidine value (AnV), total chloride and fatty acid composition (FAC) with control oil (POo). The results also showed that the presence of chloride in POo did not onset further formation of 3-MCPD esters and glycidyl esters throughout the frying period. As the behaviours of 3-MCPD esters and glycidyl esters were affected by PGE, only a sufficient amount should be added into POo to ensure oil clarity at a realistic period.

## 1. Introduction

Palm olein (POo), which is the liquid fraction of palm oil, is extensively used in commercial cooking and frying attributed by its resistance against high temperatures [1], as well as consistent supply and price competitiveness [2]. As POo has equivalent amounts of saturated and unsaturated fatty acids, a natural occurrence of solid precipitation is expected particularly when the oil is stored at a lower temperature. Nonetheless, there are consumers who still perceive cloudy oil as a deteriorated or poor-quality oil, and hence preclude them from choosing POo [3]. Similar to coconut oil, POo also experiences crystallisation when the oil is shelved in temperate countries. Incorporation of anti-clouding agent like polyglycerol fatty acid esters (PGE) has proven to delay cloudiness by slowing down crystallisation and thus improving the oil clarity [4,5].

The PGE is a class of emulsifier which is applicable in formulating solid fat products (i.e., margarines and shortening), confections (i.e., chocolate and ice-cream), and bakery products (i.e., bread and cake). It exhibits interfacial characteristics when hydrophilic and hydrophobic moieties were coexisting within the same molecules [6]. In principal, this additive is synthesised through esterification between glycerol and fatty acids of edible oil. The polarity of emulsifier is often expressed as hydrophilic-lipophilic balance (HLB) value [7]. Unlike any other food emulsifiers, PGE has a broader range of polarity or HLB values between 6 and 11. The polarity of this additive is influenced by the extent of glycerol polymerisation (ranging from monoester to polyester) and types of esterified fatty acids. Greater HLB value indicates that the emulsifier is predominantly hydrophilic in nature while emulsifier with lower HLB value is driven towards hydrophobic behaviour [5]. 

Over the last five years, thirteen frying bibliography have reported the behaviours of 3-monochloropropane-1,2-diol (3-MCPD) esters and/or glycidyl esters in edible oils, mainly in palm-based oils, when subjected to different frying conditions [8,9,10,11,12,13,14,15,16,17,18,19,20]. Issues associated to 3-MCPD esters and glycidyl esters in refined vegetable oils and fats have gained much attention in the past 10 years considering their potential health implications to humans. According to European Food Safety Authority (EFSA), the 3-MCPD esters are classified as nephrotoxic while the glycidol esters are categorised as probably carcinogenic to humans [21]. 

The European Commission recently published a Regulation (EU) 2018/290 concerning the maximum level of glycidyl esters in food [22]. This regulation has already entered into force and establishes the maximum limits of glycidyl esters in vegetable oils and fats (1 mg kg^−1^), infant formula (0.5 mg kg^−1^), and follow-on formula and food for special medical purposes intended for infants and young children (0.006 to 0.075 mg kg^−1^). The limits for 3-MCPD esters have not been established to date; nevertheless, the EU will certainly enforce the legislative limit for 3-MCPD esters in the coming years.

It is also important to note that exposing the oils to excessive heating in the presence of moisture and air can lead to multiple series of unwanted reactions such as hydrolysis, oxidation and polymerisation. These reactions are getting complicated when oil breakdown components interact with food and subsequently modify the characteristics of the oil [23]. Indeed, the degree of polarity in PGE might affect the thermal properties of POo when subjected to frying.

Due to health concern associated to heated oils, we report the fates of 3-MCPD esters and glycidyl esters, and the physico-chemical changes occurring in POo upon repeated frying when the oil is fortified with PGE at different dosages, i.e., 0.1%, 0.2%, 0.3% and 0.4%. It is speculated that the presence of PGE in POo influences the reduction of 3-MCPD esters and glycidyl esters, as well as some quality parameters when subjected to frying.

## 2. Materials and Methods

### 2.1. Raw Materials

Refined, bleached and deodorised POo was purchased from Lam Soon Edible Oils Sdn Bhd (Telok Panglima Garang, Malaysia) while Ramly Food Processing Sdn Bhd (Kuala Lumpur, Malaysia) supplied the frozen pre-fried French fries. The PGE was obtained from Global Specialty Ingredient Sdn Bhd (Pulau Indah, Malaysia) under a commercial name of HIFED MF-18.

A total of four frying media were prepared by incorporating four dosages of PGE i.e., 0.1%, 0.2%, 0.3% and 0.4% (weight basis) PGE in POo. Frying using POo without PGE was constituted as control experiment. Both POo and PGE were heated at 60 °C and 80 °C, prior to mixing to ensure homogeneity.

### 2.2. Frying Experiments

Repeated or intermittent frying experiments were performed using two units of 10-kg capacity stainless steel electrical open fryers (MSM Equipment Manufacturer Sdn Bhd, Seri Kembangan, Malaysia) with two split pots for each fryer. Five kilograms of oil was first poured into each pot and conditioned at 180 °C for 30 min prior to frying. A batch of 150 g of pre-fried French fries was fried for 3.5 min followed by 26.5 min interval before the next cycle. Sixteen frying cycles were conducted daily (8 h day^−1^) and lasted for 5 consecutive days. Five hundred grams of oil was sampled at the end of each day using a 500 mL dark amber bottle. All samples were flushed with nitrogen and stored at −20 °C for subsequent physico-chemical analyses. French fries were sampled at the 8th frying cycle on a daily basis for oil content measurement. The lid was placed on the fryer and left overnight. Daily replenishment of 500 g of fresh oil was done for each pot to replace oil sample collected for subsequent analyses.

### 2.3. Analysis of 3-MCPD Esters and Glycidyl Esters

The 3-MCPD esters and glycidyl esters were quantified simultaneously with reference to the AOCS Official Method Cd 29a-13 [24] using a single-quadrupole Gas Chromatography with Mass Spectrometer (GC-MS) (Agilent Technologies, Santa Clara, CA, USA). About 100 mg oil aliquot was first dissolved in 50 µL mixture containing tetrahydrofuran (THF) (Merck, Darmstadt, Germany) and two internal standards, i.e., 1,2-dipalmitoyl-3-chloropropanediol (PP-3-MCPD-d5) and glycidyl palmitate (Gly-P-d5) (Toronto Research Chemical, North York, Canada). The solution was then added with 30 µL of sodium bromide (Merck, Darmstadt, Germany) in 5% sulphuric acid (Merck, Darmstadt, Germany), vigorously shaken and allowed to incubate at 50 °C for 15 min. The reaction was terminated when 3 mL of 0.6 % sodium hydrogen carbonate aqueous solution was added into the sample solution, followed by 2 mL of n-heptane (Merck, Darmstadt, Germany) to separate the oil from the water phase; sodium hydrogen carbonate was obtained from (Sigma-Aldrich, St. Louis, MO, USA). The upper layer—which refers to the oil phase—was transferred into a separate glass tube and dried under nitrogen stream. The remnant was further dissolved in 1 mL of THF and subsequently added with 1.8 mL sulphuric acid in methanol solution before underwent for 16 h of incubation at 40 °C. The reaction was stopped by adding 0.5 mL of sodium hydrogen carbonate saturated solution and allow the solution to mix before removal of organic solvent under continuous flow of nitrogen. Sodium sulphate solution sodium sulphate was purchased from Merck (Darmstadt, Germany) and n-heptane of 2 mL each were then added to the remnant, followed by centrifugation to allow immediate separation of two layers. The lower layer was isolated and homogenised in 250 µL of phenylboronic acid solution (Sigma-Aldrich, St. Louis, MO, USA), incubated for 5 min using an ultrasonic water bath at ambient temperature (24 °C). Extraction of phenylboronic derivatives of 3-MCPD, and 3-monobromopropanediol (3-MBPD) were made by taking the upper layer upon mixing with 1 mL of n-heptane. The supernatant was dried under continuous nitrogen stream, dissolved in 400 µL of n-heptane and decanted for analysis using a GC-MS. 

The GC-MS was equipped with a quadrupole detector, electronic integrator and data processor. Helium gas was used as a carrier at the flow rate of 0.8 mL min^−1^ with sample injection of 1 uL set at pulsed splitless mode. A HP-5MS of 5%-phenyl-methyl-siloxane capillary column (30 m ° 0.25 mm i.d., 0.25 μm f.t.; Agilent J&W, Santa Clara, CA, USA) was fitted to the GC-MS. The quadrupole detector and injector temperature were fixed at 150 °C and 250 °C, respectively. The column was initially programmed at 80 °C and held constant for 1 min before heating to 170 °C at a rate of 10 °C min^−1^. The column temperature was then increased to 200 °C at a rate of 3 °C min^−1^, and followed by rapid heating to 300 °C at a rate of 15 °C min^−1^. Quantitative analysis was accomplished by examining the quantifier ions of the following components: (1) derivatized 3-MCPD at *m*/*z* 147, 196 and 198; (2) 3-MCPD-d_5_ at *m*/*z* 150, 201 and 203; (3) derivatized 3-MBPD at *m*/*z* 147 and 240; and (4) 3-MBPD-d_5_; at *m*/*z* 150 and 245. The 3-MCPD esters and glycidyl esters peaked within 20 min of analysis time. The recovery for different concentrations of 3-MCPD esters and glycidyl esters ranged between 80.3% and 107.7%. The relative standard deviation (RSD) of the replicates was within 2.9 and 13.7, which suggests good repeatability and reproducibility of the test performed. Limit of detection (LOD) for the analytes were established at 0.02 mg kg^−1^ for 3-MCPD esters and 0.05 mg kg^−1^ for glycidyl esters. Limit of quantitation is calculated as 10 x LOD of which 0.2 mg kg^−1^ for 3-MCPD esters and 0.5 mg kg^−1^ for glycidyl esters.

### 2.4. Analysis of Chloride Content

The amount of chloride in fresh and used oils was measured using a Total Chlorine Analyzer (TCA) NSX-2100H (Mitsubishi Chemical Analytech, Kanagawa, Japan) following the method described in ASTM D4929-04 [25]. The operation of TCA underpins the principle of combustion and microcoulometry. In oil aliquot, there was 65 mg combusted in the presence of argon and oxygen at a temperature range of 800 and 1000 °C. The hydrogen chloride released as a result of combustion was then titrated with silver ion emitted from the titration cell. Chloride content is quantified based on the intensity of electrical current release in the microcoulometer throughout the titration stage. 

### 2.5. Analysis of Total Polar Compounds

Gravimetric method was used to measure total polar compounds (TPC) and their fractions following IUPAC 2.507 [26] with some modifications [27]. There was 1 g of oil aliquot initially dissolved in 20 mL of mixture containing 87% petroleum ether (PE) and 13% diethyl ether (DE); both solvents were supplied by Systerm (Shah Alam, Malaysia). The solution was then transferred into a glass chromatography column packed with 25 g of Silica Gel 60 No. 7734 (Merck, Darmstadt, Germany), suspended in the PE-DE mixture and covered with 4 g of sea sand. The non-polar fraction was first eluted with 150 mL of PE-DE at the flow rate of 2.5 mL min^−1^, followed by 150 mL of PE at similar flow rate to isolate polar fraction. Removal of solvent from polar and non-polar solutions was made using a rotary evaporator (Büchi Labortechnik AG, Flawil, Switzerland) for 15 min at water temperature of 60 °C, vacuum level of 380 mbar and rotation speed of 130 rpm. In order to ensure that the solvent residue was completely removed, the sample underwent subsequent drying under nitrogen stream for 15 min so that constant weight can be achieved.

### 2.6. Analysis of Polar Compound Fractions

Partitioning of polar compounds were further examined using a High-Performance Size Exclusion Chromatography (HPSEC) equipped with Evaporative Light Scattering Detector (ELSD) and three PLgel columns of 500 Ǻ, 5 μm particle size with the dimension of 7.5 mm × 300 mm (Agilent 1260 Infinity, Santa Clara, USA). A mixture of 5 mg mL^−1^ sample in THF (Merck, Darmstadt, Germany) was prepared and filtered with a 0.45 μm nylon membrane filter prior to instrument analysis. THF was used as the mobile phase and fixed at the flow rate of 1 mL min^−1^. All columns and ELSD evaporator were set at 40 °C while ELSD nebulizer was held at 30 °C. Nitrogen stream for ELSD was positioned at 1.7 standard L min^−1^ (SLM). A complete run for each sample required about 30 min of analysis.

### 2.7. Other Analytical Tests

Free fatty acid (FFA) and *p*-anisidine value (AnV) were analysed using the AOCS Official Methods Ca 5a-40 and Cd 18-90, respectively [24]. The fatty acid methyl ester (FAME) was prepared following the AOCS Official Method Ce 1i-07 [24]. A Hewlett-Packard 6890 Series GC equipped with a DB-23 fused silica capillary column (60 m × 0.25 mm, i.d. 0.25 μm film thickness), flame ionisation detector (FID), electronic integrator and data processor (J & W Scientific, Folsom, CA, USA) was used to measure the fatty acid composition (FAC). The procedure for FAC analysis was performed following Ahmad Tarmizi et al. [27]. Oil content was determined according to AOAC Official Method 945.16 [28].

### 2.8. Data Evaluation

All results presented in this study were expressed as mean ± standard deviation of duplicate frying experiments while each analytical measurement was conducted in triplicate. Data arrangement and figure illustration were performed using Microsoft Office Excel 2007 (Redmond, WA, USA). Data generated at different treatments were compared by one-way analysis of variance (ANOVA) using SPSS software (SPSS Statistics 21, IBM Corp., Armonk, NY, USA). Tukey test was used to evaluate the significant differences between means at *p* < 0.05 while relationships between quality and safety parameters were determined using Pearson correlation test. 

## 3. Results and Discussion

### 3.1. Transients of 3-MCPD Esters and Glycidyl Esters during Repeated Frying

**Figure 1:**
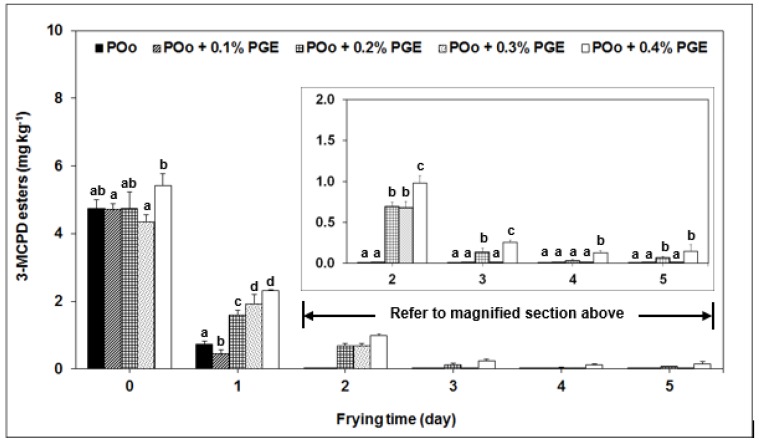
Degradation of 3-MCPD esters in palm olein (Poo) added with polyglycerol fatty acid esters (PGE) during repeated frying. The 3-MCPD esters for frying times between Day-2 and Day-5 can be referred to the magnified section to improve clarity. One-way analysis of variance (ANOVA) was used to indicate the significant difference between the control experiment (POo without PGE) and each frying interval (*p* < 0.05), as shown by different lowercase letters.

Figure 1 illustrates the transient of 3-MCPD esters in POo containing PGE as a function of frying time. The initial levels of 3-MCPD esters (Day-0) for all treatments were insignificantly different when compared to POo without PGE. The results also showed that 40 h of intermittent frying gave drastic descending trend of 3-MCPD esters just after 8 h of heating and continued to depreciate gradually thereafter. This prevalence is more apparent in the controlled oil and POo fortified with least amount of PGE (0.1%); the concentrations of 3-MCPD esters in both oils have levelled off at 0.01 mg kg^−1^ after 2 days of frying. Increase the PGE dosages of more than 0.1% retarded the decomposition rate of 3-MCPD esters even though the final concentration were considerably low at the end of frying session (0.01 to 0.15 mg kg^−1^).

In principle, the overall level of 3-MCPD esters in used oil reached a state of equilibrium between the decomposition of existing 3-MCPD esters and the formation of new 3-MCPD esters. In this study, heating the oil at high frying temperature for an extended time prevailed a prominent reduction of 3-MCPD esters since they are unstable and have tendency to disintegrate. Slower decomposition rate of 3-MCPD esters in POo fortified with PGE can be explained from the basis of higher developments of polar compound fractions, which are oxidised triacylglycerols (OxTAG) and polymerised triacylglycerols (PTAG) upon frying (Table 1). According to Hamlet et al. [29], depreciation of TAG, as a result of TAG breakdowns, leads to the formation of intermediate acyloxonium ions, which further promotes the formation of 3-MCPD esters. However, this observation was only applicable for the first two days of frying as 3-MCPD esters appeared to be comparatively low thereafter (Figure 1).

From our observation, we hypothesised that the endogenous formation of new 3-MCPD esters can be considered insignificant in all heated oils as their chloride contents appeared identical to that of fresh oils (Table 2). Slim variation in chloride content (2 and 3 mg kg^−1^) is probably due to some of the chloride in the French fries migrating to the oil during frying. It is also worth noting that the presence of natural chloride in potato (739 mg kg^−1^) as reported by Arisseto et al. [14] did not onset further formation of 3-MCPD esters.

Our findings, however, contradicted with the work done by Wong et al. [13] where 3-MCPD esters continued to build-up during frying of chicken meat containing sodium chloride (1% to 5%) Excessive amount of sodium chloride of up to 50,000 mg kg^−1^ (5%) provides adequate chloride ions to generate new 3-MCPD esters through interaction with acylglycerols via acyloxonium ions [30], which in turn, surpassed the amount of decomposed 3-MCPD esters. However, immersion of potato slices in sodium chloride exhibited a gradual drop of 3-MCPD esters upon frying albeit the presence of sodium chloride seemed to delay 3-MCPD esters degradation [12]. We speculate that food structure, diffusivity of sodium chloride in food and/or amount of sodium chloride adhered on the food surface are likely to affect the transition of overall 3-MCPD esters in the oil; however, this will require further investigation and confirmation.

**Figure 2:**
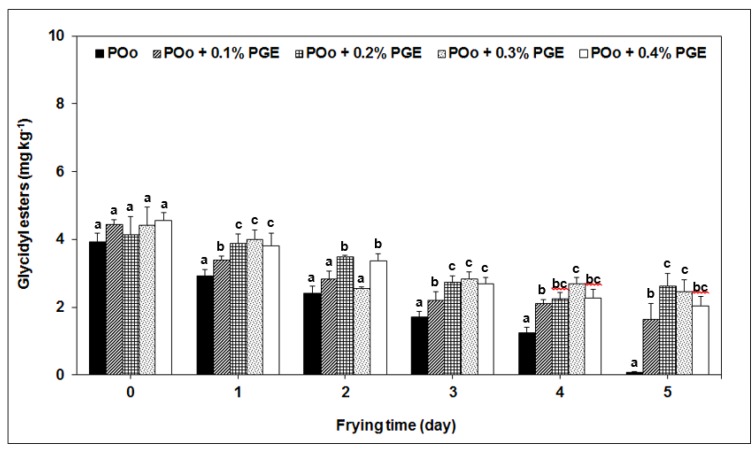
Reduction of glycidyl esters in POo added with PGE during repeated frying. One-way analysis of variance (ANOVA) was used to indicate the significant difference between the control experiment (POo without PGE) and each frying interval (*p* < 0.05) as showed by different lowercase letters.

In the case of glycidyl esters, the trend went down steadily over the frying period (Figure 2). It is obvious that the PGE slowed down the loss of glycidyl esters in POo owing their high retention of initial glycidyl esters between 39% and 56% after five days of frying. Conversely, the absence of PGE in POo exhibited remarkable drop of glycidyl esters content of nearly 98% at the end of frying session. Similar to 3-MCPD esters, our study confirmed that degradation of existing glycidyl esters was higher than the newly developed glycidyl esters in used oils [9,10,19]. Indeed, glycidyl esters can even experience destabilisation when oil is subjected to high frying temperature. According to Hamlet et al. [29], epoxides are very reactive artefacts of which the epoxide ring can be opened by different nucleophiles—i.e., acids, alcohols, water, amines and thiols—under acidic and alkaline conditions. It is fully understood that diacylglycerols (DAG) is the precursor for the formation of glycidyl esters in oil [31]. Despite the amount of DAG seemed identical in all tested oils, the reduction of glycidyl esters was noticeable in the controlled oil (POo). It is plausible that the polarity of PGE can be the possible reason for slow reduction of glycidyl esters. Nevertheless, two studies by Wong et al. [12,13] displayed an upward trend in glycidyl esters throughout the frying course. They believed that endogenous formation of new glycidyl esters is more prevalence than the disintegration of glycidyl esters. 

In this study, we also measured the amount of oil being taken up the fried product. Regardless to the treatments applied, the oil contents in finished product (French fries) varied between 12% and 16% when the product was sampled daily across 5 days of frying. By taking 14% as the average oil content, one can be conjecture that the amounts of 3-MCPD esters and glycidyl esters were no higher than 0.32 mg kg^−1^ and 0.56 mg kg^−1^, respectively just after frying for 1 day, and these values continued to lessen thereafter particularly for 3-MCPD esters. The result indicates that finish frying of French fries would contain lower levels of 3-MCPD esters and glycidyl esters. Similar observation holds true with the findings by Arisseto et al. [14] where the 3-MCPD esters and glycidyl esters contents in finished products can be predicted from their oil contents. Of course, products with higher surface-to-volume ratio absorb more oil and most likely have higher levels of 3-MCPD esters and glycidyl esters. 

### 3.2. Thermal Resistance of Oils during Repeated Frying

Quantification of TPC is often regarded as an objective method to distinguish the degree of oil deterioration in used oils. Nevertheless, TPC measurement can sometimes be deceptive particularly for those oils with higher diacylglycerols (DAG) content [27]. Our study, therefore, did not limit to the measurement of TPC alone, but also identified the individual fractions of polar compounds namely as DAG, OxTAG and PTAG. The progressions of TPC in POo added with PGE are displayed in Table 1. The initial TPC in POo before and after fortification of PGE varied within a narrow range of 7 and 8% of which nearly 90% of the total TPC is constituted by DAG. The impact of PGE was more apparent when the oils were subjected for 40 h of frying. At the end of frying operation, it was observed that the PGE content affects TPC development in POo. As expected, inclusion of PGE in POo literally enhanced the formation of TPC; nevertheless, TPC started to plateau when PGE exceeds 0.2%. From Table 1, it is also noted that all oils exceeded the TPC cut-off point of 27% after 3 days of frying [32]. The rate of TPC was lowest in POo without additive (4.93% day^−1^) while increasing the PGE dosage fastened the TPC formation between 5.22 day^−1^ and 5.51% day^−1^. Except for DAG, the formation rate of other polar fractions, i.e., OxTAG and PTAG, were influenced by the increase in PGE dosage. It is reported that the threshold for PTAG can be as low as 10% [33].

We also gauged the acidity of oils based on the formation of free fatty acid (FFA). Hydrolytic reaction is the predominant cause of FFA development when oil interacts with the moisture released from food even though FFA can be also generated through breakdown of triacylglycerol (TAG) when reacted with air (oxygen) [34]. The result depicted in Table 2 indicates that the acidity in blank POo and POo fortified with different dosages of PGE increased throughout the frying course. Regardless to any treatments performed, the amount of FFA developed in all oils was considered low even after 5 days of frying by taking the legal limits of FFA within 1 and 3% depending to countries [35].

Peroxide value (PV) generally measures the presence of hydroperoxides in oil during early stage of oxidation. Nevertheless, PV becomes irrelevant when the oil is subjected under frying conditions because hydroperoxides have tendency to breakdown upon intense heating [36]. Hence, the measurement of *p*-anisidine value (AnV) is more suitable for evaluating the prevalence of oxidation during frying since the aldehydes developed are more stable than hydroperoxides [37]. The initial AnV remained lower than 3 unit in all oils which further attest that incorporation of PGE did not adversely affect their oxidative stability. Regardless to PGE concentrations, the AnV increased rapidly just after one day of frying and subsequent gradual increase throughout the remaining frying times (Table 2). Interaction between oil and air (oxygen) under extended frying conditions will establish carbon-to-carbon linkages between TAG molecules and therefore lead to the formation of higher molecular weight compounds [38]. Similar to FFA, the AnV was insignificantly different between control oil (POo) and POo added with PGE upon completion of frying. It is important to mention that slight disparity trends in OxTAG and AnV is expected since AnV analysis only measure the degree of oxidation based on the presence of aldehyde alone and omits the formation of ketonic compounds [39]. 

In principal, FAC is one the indicators used to estimate thermal stability of the oil. Table 3 shows the FAC of fresh and used POo after 5 days of frying. Inclusion of PGE at any concentrations did not affect the initial and final FAC of POo even after 40 h of heating. In fact, the C18:2/C16:0 ratio—which measures the degree of oil to undergo oxidative breakdown—seemed identical before (0.35) and after the frying session (between 0.19 and 0.20). As expected, the changes occurring in FAC was more pronounced when prolonging the heating period. Increase in the oil temperature also accelerates the alteration of fatty acids [10]. It is important to note that the apparent increase in palmitic acid (C16:0) and stearic acid (C18:0) after frying is due to normalisation of fatty acids peaks and not resulted from newly developed fatty acids. Qualitative elevation in the values of C16:0 and C18:0 is caused by severe breakdowns of unsaturated fatty acids particularly for linoleic acid (C18:2), which encountered between 35% and 38% reduction at the end of frying session. This significant degradation is associated to the relative oxidation rate of C18:2 with 100 times higher than that of C18:0 due to the presence of two double bonds in its structure [40]. Systematic studies by Giua et al. [41] and Cossignani et al. [42] showed that disintegration of C18:2 into hydroperoxides, isomers and volatile organic compounds (i.e., aldehydes, alcohols, furans, methyl esters and methyl-oxoacids) very much relies on the chemical forms of this fatty acid, which can be either FFA, methyl esters and/or homogeneous TAG. 

### 3.3. Assessments of 3-MCPD Esters and Glycidyl Esters with Quality Parameters

Pearson correlation test was used to identify linear relationship between heat-induced contaminants (i.e., 3-MCPD esters and glycidyl esters) and selected quality parameters. The strength of relationship between two variables tested is expressed as correlation coefficient (r). All parameters exhibited negative and strong correlations with 3-MCPD esters (r = 0.80 to r = 0.96) (Table 4).

Similar to 3-MCPD esters, glycidyl esters also yielded negative correlation between all parameters. Strong correlation was detected between glycidyl esters, and TPC, OxTAG, PTAG and FFA (r ≥ 0.80) whereas, DAG and AnV showed moderate correlation with glycidyl esters. Aniołowska and Kita [10] obtained nearly perfect positive correlation between glycidyl esters and DAG, while correlations between glycidyl esters and other parameters (i.e., TPC, OxTAG and PTAG) were negative and almost perfect when single type of oil was used for frying. Comparing different types of frying medium gave moderate correlation between glycidyl esters and quality parameters [9].

## 4. Conclusions

This study showed that mixing PGE in POo affects the behaviours of 3-MCPD esters and glycidyl esters during excessive frying. Progressive drop of 3-MCPD esters was only noticeable in POo containing none or the least amount of PGE (0.1%). Slower deformation of 3-MCPD esters in POo with higher PGE dosages can be linked to an increase in polar compound fractions. Higher degree of TAG degradation induced the development of new 3-MCPD esters during frying, and subsequently moderates the overall reduction of 3-MCPD esters in the oils. Chloride content seemed plateauing across frying times irrespective to oil samples, which clearly showed that endogenous formation of new 3-MCPD esters is unimportant. Blank POo exhibited notable drop in glycidol esters especially after five days of frying; whereas the inclusion of PGE delayed the degradation of glycidyl esters. Irrespective to PGE dosages, there was no distinctive different in FFA, AnV, total chloride and FAC when compared to the control oil. It is therefore recommended that inclusion of PGE in POo is not required unless the oil is stored at temperate conditions. Moreover, the amount of PGE added should be optimised and sufficient to allow the oil remains clear at realistic storage period.

## Figures and Tables

**Table 1:** Changes of quality parameters in palm olein (Poo) added with polyglycerol fatty acid esters (PGE) during repeated frying.

Parameters	Treatments	Frying Time (Day)					
		0	1	2	3	4	5
TPC (%)	POo	7.09 ± 0.47 ^a,A^	14.46 ± 0.69 ^a,B^	22.75 ± 0.04 ^a,C^	27.84 ± 0.54 ^a,D^	31.32 ± 0.02 ^a,E^	31.76 ± 0.27 ^a,F^
	POo + 0.1% PGE	6.98 ± 0.88 ^a,b,A^	15.13 ± 0.22 ^a,B^	23.04 ± 0.28 ^a,C^	28.13 ± 1.10 ^a,b,D^	31.73 ± 0.54 ^a,b,E^	33.41 ± 0.29 ^b,F^
	POo + 0.2% PGE	8.03 ± 0.35 ^b,A^	16.26 ± 0.67 ^b,B^	23.50 ± 0.11 ^b,C^	28.03 ± 0.46 ^a,D^	32.06 ± 0.22 ^b,E^	34.15 ± 0.08 ^c,F^
	POo + 0.3% PGE	7.18 ± 0.34 ^a,A^	15.39 ± 0.10 ^a,B^	23.69 ± 0.33 ^b,C^	29.31 ± 0.11 ^b,D^	31.95 ± 0.39 ^a,b,E^	34.79 ± 0.21 ^d,F^
	POo + 0.4% PGE	7.83 ± 0.78 ^a,b,A^	16.64 ± 1.12 ^b,B^	23.49 ± 0.04 ^b,C^	29.32 ± 1.03 ^a,b,D^	32.24 ± 0.93 ^a,b,E^	34.47 ± 1.04 ^b,c,d,F^
DAG (%)	POo	6.18 ± 0.32 ^a,A^	7.92 ± 0.43 ^a,B^	8.22 ± 0.07 ^a,B^	8.28 ± 0.52 ^a,B,C^	8.40 ± 0.05 ^a,B^	8.62 ± 0.11 ^a,C^
	POo + 0.1% PGE	6.30 ± 0.52 ^a,b,A^	7.84 ± 0.21 ^a,B^	8.01 ± 0.06 ^b,B^	7.96 ± 0.27 ^a,B,C^	8.32 ± 0.13 ^a,b,C,D^	8.53 ± 0.14 ^a,D^
	POo + 0.2% PGE	7.03 ± 0.25 ^b,A^	8.22 ± 0.28 ^a,B^	7.82 ± 0.06 ^c,C^	8.33 ± 0.13 ^a,B^	8.19 ± 0.07 ^b,B^	8.83 ± 0.23 ^a,D^
	POo + 0.3% PGE	6.50 ± 0.38 ^a,b,A^	8.44 ± 0.45 ^a,B,C^	8.16 ± 0.06 ^a,B^	8.53 ± 0.53 ^a,B,C^	8.35 ± 0.14 ^a,b,B^	9.01 ± 0.33 ^a,C^
	POo + 0.4% PGE	6.73 ± 0.58 ^a,b,A^	8.05 ± 0.55 ^a,B,C^	7.90 ± 0.08 ^b,c,B^	8.01 ± 0.68 ^a,B,C^	8.25 ± 0.17 ^a,b,C^	8.87 ± 0.50 ^a,C^
OxTAG (%)	POo	0.67 ± 0.18 ^a,A^	3.79 ± 0.01 ^a,B^	7.95 ± 0.04 ^a,C^	10.42 ± 0.49 ^a,D^	11.31 ± 0.09 ^a,E^	10.14 ± 0.61 ^a,D^
	POo + 0.1% PGE	0.51 ± 0.13 ^a,b,A^	4.03 ± 0.38 ^a,b,B^	8.07 ± 0.38 ^a,d,C^	10.70 ± 1.49 ^a,b,D^	11.57 ± 0.51 ^a,b,D^	10.81 ± 0.61 ^a,b,D^
	POo + 0.2% PGE	0.56 ± 0.05 ^a,A^	4.48 ± 0.09 ^b,c,B^	8.71 ± 0.06 ^b,C^	10.34 ± 0.52 ^a,D,E^	11.92 ± 0.18 ^b,D^	10.53 ± 0.30 ^a,E^
	POo + 0.3% PGE	0.35 ± 0.04 ^b,A^	3.83 ± 0.30 ^a,B^	8.39 ± 0.20 ^d,C^	10.00 ± 0.59 ^a,D^	11.69 ± 0.10 ^b,E^	11.30 ± 0.08 ^b,F^
	POo + 0.4% PGE	0.90 ± 0.39 ^a,A^	5.05 ± 0.61 ^c,B^	8.55 ± 0.18 ^d,C^	11.83 ± 0.43 ^b,D^	11.94 ± 0.30 ^b,D^	11.80 ± 0.67 ^b,D^
PTAG (%)	POo	0.10 ± 0.01 ^a,A^	2.61 ± 0.20 ^a,B^	6.52 ± 0.06 ^a,C^	8.56 ± 0.17 ^a,D^	11.53 ± 0.10 ^a,E^	12.60 ± 0.49 ^a,F^
	POo + 0.1% PGE	0.04 ± 0.01 ^b,A^	3.10 ± 0.27 ^b,B^	6.89 ± 0.13 ^b,d,C^	9.38 ± 0.37 ^b,D^	11.78 ± 0.08 ^b,c,E^	13.55 ± 0.46 ^a,b,F^
	POo + 0.2% PGE	0.22 ± 0.02 ^c,A^	3.27 ± 0.31 ^b,B^	6.89 ± 0.08 ^b,d,C^	9.30 ± 0.16 ^b,D^	11.88 ± 0.08 ^b,E^	13.98 ± 0.03 ^b,F^
	POo + 0.3% PGE	0.18 ± 0.03 ^c,A^	2.95 ± 0.06 ^b,B^	7.10 ± 0.04 ^c,C^	9.16 ± 0.05 ^b,D^	11.84 ± 0.13 ^b,c,E^	14.44 ± 0.16 ^c,E^
	POo + 0.4% PGE	0.12 ± 0.09 ^a,c,A^	3.02 ± 0.25 ^a,b,B^	7.01 ± 0.07 ^c,d,C^	9.29 ± 0.57 ^a,b,D^	12.00 ± 0.30 ^c,E^	14.21 ± 0.30 ^b,c F^

Means within a row for each parameter marked with the same lowercase letters are insignificantly different (*p* < 0.05). Means within a column for each parameter marked with the same uppercase letters are insignificantly different (*p* < 0.05). Abbreviations: TPC—total polar compounds; DAG—diacylglycerols; OxTAG—oxidised triacylglycerols; PTAG—polymerised triacylglycerols.

**Table 2:** Changes of FFA, Totox value and total chlorine in POo added with PGE during repeated frying.

Parameters	Treatments	Frying Time (Day)					
		0	1	2	3	4	5
FFA (%)	POo	0.10 ± 0.01 ^a,A^	0.29 ± 0.06 ^a,B^	0.48 ± 0.08 ^a,C^	0.66 ± 0.11 ^a,C^	0.84 ± 0.04 ^a,D^	0.95 ± 0.12 ^a,D^
	POo + 0.1% PGE	0.10 ± 0.02 ^a,A^	0.26 ± 0.06 ^a,B^	0.42 ± 0.09 ^a,C^	0.58 ± 0.11 ^a,C,D^	0.73 ± 0.09 ^a,D^	0.87 ± 0.04 ^a,E^
	POo + 0.2% PGE	0.13 ± 0.05 ^a,A^	0.29 ± 0.07 ^a,B^	0.45 ± 0.07 ^a,C^	0.60 ± 0.09 ^a,C,D^	0.75 ± 0.09 ^a,D,E^	0.89 ± 0.08 ^a,E^
	POo + 0.3% PGE	0.09 ± 0.01 ^a,A^	0.25 ± 0.09 ^a,B^	0.43 ± 0.02 ^a,C^	0.59 ± 0.06 ^a,D^	0.76 ± 0.08 ^a,D^	0.91 ± 0.09 ^a,E^
	POo + 0.4% PGE	0.09 ± 0.01 ^a,A^	0.29 ± 0.07 ^a,B^	0.47 ± 0.10 ^a,C^	0.65 ± 0.02 ^a,D^	0.83 ± 0.05 ^a,E^	0.98 ± 0.06 ^a,F^
AnV (unit)	POo	1.67 ± 0.22 ^a,A^	51.56 ± 6.41 ^a,B^	61.23 ± 7.86 ^a,B^	61.23 ± 6.86 ^a,B^	62.06 ± 5.15 ^a,B^	68.71 ± 4.97 ^a,C^
	POo + 0.1% PGE	1.40 ± 0.10 ^a,A^	59.25 ± 2.25 ^a,B^	63.69 ± 1.64 ^a,C^	66.91 ± 5.72 ^a,B,C^	69.28 ± 5.39 ^a,C^	68.62 ± 4.96 ^a,C^
	POo + 0.2% PGE	2.23 ± 0.22 ^b,A^	52.59 ± 7.84 ^a,B^	59.62 ± 6.26 ^a,B^	59.88 ± 7.91 ^a,B^	58.33 ± 6.36 ^a,B^	63.65 ± 5.73 ^a,B^
	POo + 0.3% PGE	1.71 ± 0.32 ^a,b,A^	52.20 ± 7.22 ^a,B^	62.59 ± 6.92 ^a,B^	62.36 ± 5.17 ^a,B^	61.23 ± 5.99 ^a,B^	64.93 ± 7.73 ^a,B^
	POo + 0.4% PGE	1.40 ± 0.22 ^a,A^	57.02 ± 2.26 ^a,B^	64.64 ± 5.10 ^a,B,C^	64.60 ± 3.60 ^a,C^	68.28 ± 5.17 ^a,C^	67.71 ± 4.39 a^,C^
Chloride content (mg kg^−1^)	POo	2.37 ± 0.09 ^a,c,A,B^	2.14 ± 0.20 ^a,A^	2.36 ± 0.00 ^a,A^	2.46 ± 0.05 ^a,B^	2.52 ±0.07 ^a,B^	2.50 ± 0.15 ^a,A,B^
	POo + 0.1% PGE	2.41 ± 0.13 ^a,c,A,B^	2.31 ± 0.09 ^a,A^	2.32 ± 0.12 ^a,A,B^	2.38 ± 0.04 ^a,A^	2.38 ± 0.03 ^b,A^	2.58 ± 0.12 ^a,B^
	POo + 0.2% PGE	2.18 ± 0.15 ^a,A^	1.77 ± 0.09 ^b,B^	1.94 ± 0.08 ^b,A^	2.02 ± 0.20 ^b,A,B^	2.01 ± 0.19 ^c,A,B^	2.02 ± 0.15 ^b,A,B^
	POo + 0.3% PGE	2.68 ± 0.02 ^b,A^	2.71 ± 0.20 ^c,A,B^	2.75 ± 0.03 ^c,A,B^	2.79 ± 0.18 ^c,A,B,C^	2.90 ±0.17 ^d,B,C^	3.09 ± 0.12 ^c,C^
	POo + 0.4% PGE	2.58 ± 0.11 ^b,c,A^	2.70 ± 0.01 ^c,A^	2.79 ± 0.04 ^c,A^	2.93 ± 0.03 ^c,B^	2.97 ± 0.04 ^d,B^	3.16 ± 0.01 ^c,C^

Means within a row for each parameter marked with the same lowercase letters are insignificantly different (*p* < 0.05). Means within a column for each parameter marked with the same uppercase letters are insignificantly different (*p* < 0.05). Abbreviations: FFA—free fatty acid; AnV—*p*-anisidine value.

**Table 3:** Changes of FAC in POo added with PGE during repeated frying.

Treatments	Frying Time (Day)	FAC (%)					
		C16:0	C18:0	C18:1	C18:2	C18:3	C18:2/C16:0
POo	0	35.25 ± 0.05 ^a^	3.31 ± 0.02 ^a^	46.65 ± 0.04 ^a^	12.38 ± 0.03 ^a,d^	0.34 ± 0.01 ^a^	0.35
	5	39.83 ± 0.03 ^b^	3.74 ± 0.01 ^b^	45.92 ± 0.04 ^b^	7.90 ± 0.00 ^b^	0.25 ± 0.08 ^a,b,d^	0.20
POo + 0.1% PGE	0	35.06 ± 0.05 ^c^	3.33 ± 0.01 ^a^	46.81 ± 0.03 ^c^	12.38 ± 0.02 ^a^	0.30 ± 0.01 ^b^	0.35
	5	39.79 ± 0.17 ^d^	3.76 ± 0.01 ^b,c^	46.27 ± 0.20 ^d^	7.68 ± 0.03 ^c^	0.12 ± 0.01 ^c^	0.19
POo + 0.2% PGE	0	35.21 ± 0.06 ^a^	3.33 ± 0.02 ^a^	46.64 ± 0.06 ^a,e^	12.36 ± 0.02 ^a,d^	0.34 ± 0.02 ^a^	0.35
	5	39.93 ± 0.08 ^d,e^	3.76 ± 0.01 ^bc^	45.86 ± 0.08 ^b,f^	7.86 ± 0.04 ^b^	0.21 ± 0.12 ^a,d^	0.20
POo + 0.3% PGE	0	35.34 ± 0.09 ^a,f^	3.33 ± 0.01 ^a^	46.51 ± 0.06 ^e^	12.34 ± 0.05 ^a,d^	0.36 ± 0.03 ^a^	0.35
	5	40.09 ± 0.08 ^e^	3.78 ± 0.01 ^c^	45.73 ± 0.05 ^f^	7.76 ± 0.02 ^e^	0.19 ± 0.01 ^d^	0.19
POo + 0.4% PGE	0	35.35 ± 0.04 ^f^	3.34 ± 0.01 ^a^	46.37 ± 0.03 ^g^	12.34 ± 0.01 ^d^	0.38 ± 0.03 ^a^	0.35
	5	39.83 ± 0.10 ^d^	3.76 ± 0.01 ^b,c^	45.73 ± 0.09 ^f^	8.02 ± 0.04 ^f^	0.25 ± 0.10 ^a,b,d^	0.20

Means within a column for each fatty acid marked with the same lowercase letters are insignificantly different (*p* < 0.05). Abbreviations: C16:0—palmitic acid; C18:0—stearic acid; C18:1—oleic acid; C18:2—linoleic acid; C18:3—linolenic acid.

**Table 4:** Significant correlation coefficients between quality parameters and 3-MCPD esters and glycidyl esters respectively.

Parameters	Significant Correlation Coefficients	
	3-MCPD Esters	Glycidyl Esters
TPC	−0.87	−0.80
DAG	−0.88	−0.70
OxTAG	−0.89	−0.80
PTAG	−0.82	−0.83
FFA	−0.80	−0.86
AnV	−0.96	−0.72

Abbreviations: TPC—total polar compounds; DAG—diacylglycerols; OxTAG—oxidised triacylglycerols; PTAG—polymerised triacylglycerols; FFA—free fatty acid; AnV—*p*-anisidine value.
